# Association between the immune-inflammation indicators and osteoarthritis - NHANES 1999–2018

**DOI:** 10.1016/j.ocarto.2024.100453

**Published:** 2024-02-29

**Authors:** Yan Xue, Cheng Chang, Yajun Chen, Lang Jia, Han Wang, Zaoyang Liu, Jiang Xie

**Affiliations:** a Department of Pediatrics, The Third People's Hospital of Chengdu, Affiliated Hospital of Southwest Jiaotong University & The Second Affiliated Hospital of Chengdu, Chongqing Medical University, Chengdu, Sichuan, China; b Department of Cardiothoracic Surgery, The Affiliated Hospital of Southwest Jiaotong University, The Third People's Hospital of Chengdu, Chengdu 610014, China; c Institute of Biomedical Engineering, College of Medicine, Southwest Jiaotong University, Chengdu 610031, Sichuan, China; d North Sichuan Medical College, Nanchong 637100, China; e School of Clinical Medicine, Southwest Medical University, Luzhou, Sichuan 646000, China; f Department of Cardiology, The Affiliated Hospital of Southwest Jiaotong University, The Third People's Hospital of Chengdu, Chengdu 610014, China

**Keywords:** Systemic immune-inflammation index, Systemic immune response index, Osteoarthritis, NHANES, ln-SII, RCS

## Abstract

**Background:**

Investigate the link between systemic immune-inflammatory index (SII) and Systemic Immune Response Index (SIRI) with osteoarthritis (OA) using National Health and Nutrition Examination Survey (NHANES) data (1999–2018).

**Methods:**

Extracted NHANES data (1999–2018) and selected a study population based on demographic, examination, and laboratory data. Calculated SII (platelet count ​× ​neutrophil count/lymphocyte count) and SIRI (neutrophil count ​× ​monocyte count/lymphocyte count). Employed multivariate logistic regression and restricted cubic spline (RCS) regression for Ln-SII, SIRI, and OA relationship investigation. Conducted subgroup analyses.

**Results:**

Study involved 32,144 participants (16,515 males, 15,629 females), with 12.16% having OA. Positive correlation between highest SII quartile and OA in unadjusted and adjusted model 1 (Unadjusted Model, P ​< ​0.001; Model 1, P ​= ​0.01). In Model 2, adjusting for all factors, positive correlation observed, not statistically significant (Model 2, P ​= ​0.07). Similar SIRI-OA correlation trends from Unadjusted Model to Model 2 (Unadjusted Model, P ​< ​0.0001; Model 1, P ​< ​0.0001; Model 2, P ​< ​0.001). Subgroup analysis found no significant factors. Identified critical point at ln-SII ≈6.39 (SII ​= ​595.86), beyond which OA prevalence significantly increased. No potential nonlinear SIRI-OA association (NL-P value ​> ​0.05).

**Conclusion:**

When SII exceeds 595.86, OA prevalence may rise. Besides, there was a significant positive correlation between SIRI and OA prevalence. SII and SIRI may be useful markers for OA research, warranting further exploration in this area.

## 1 Introduction

In the past, it was widely accepted that mechanical bone and joint damage primarily resulted from cartilage degradation [1]. However, there is a growing realization that the development and progression of osteoarthritis (OA) may involve more than just cartilage degradation; it may also encompass synovitis and chronic subchondral inflammatory conditions. OA is a prevalent joint ailment characterized by continuous articular cartilage degeneration, including chondrocyte death and extracellular matrix loss [2]. It has a high global prevalence and is a leading cause of disability, particularly among individuals aged 50 and above, often causing joint swelling, mobility difficulties, and potential permanent damage [3,4]. Unfortunately, there is currently no effective cure for OA.

Considerable evidence suggests that obesity significantly increases the risk of both developing and progressing OA [[5], [6], [7], [8]]. Some scholars speculate that obesity may play a role in OA's pathogenesis, possibly due to its association with systemic inflammation and the release of inflammatory factors. Inflammatory cytokines, in conjunction with the aging process [9], can impact chondrocytes in the body, and inflammasome activation can trigger immune responses that manifest in joint injuries [10,11]. Furthermore, a retrospective study by scholar Lee on non-surgical melanoma patients found that obesity and visceral fat index are interrelated with the systemic immune-inflammatory status determined by the systemic immune-inflammation index (SII) [12].

SII and Systemic Immune Response Index (SIRI) are novel inflammatory biomarkers that may be to evaluate systemic inflammation, primarily correlates with peripheral platelet count (P), neutrophil count (N), monocyte count (M) as well as lymphocyte count (L) [13]. The formula for calculating SII and SIRI are as follows: SII = (P ​× ​N)/L [14]. SIRI = (N ​× ​M)/L [15]. Apart from reflecting systemic inflammatory status, some scholars believe they may be associated with various diseases, including tumors [16,17], sepsis [18], ankylosing spondylitis [19], rheumatoid joint [20], and depression [21]. More commonly, it is employed to gauge disease severity, aiding in the development of prevention and treatment strategies. In this study, our aim is to use the National Health and Nutrition Examination Survey (NHANES) database to explore the association between SII, SIRI and the presence of OA.

## 2 Materials and methods

### 2.1 Study design and data source

NHANES is a comprehensive population survey database conducted in the United States. It employs a multi-stage probability sampling method to collect extensive information on nutrition, health, and various other factors related to the non-institutionalized general population in the United States. The survey is conducted biennially, gathering valuable data that helps to assess the health status and needs of the population. NHANES ensures accessibility to a significant portion of its data for academic use. The data utilized in this study are publicly available and can be accessed at http://www.cdc.gov/nchs/nhanes/index.htm. The collection of this data was conducted in compliance with the ethical review board of the National Center for Health Statistics (NCHS), and informed consent was obtained from all participants.

**Fig. 1:**
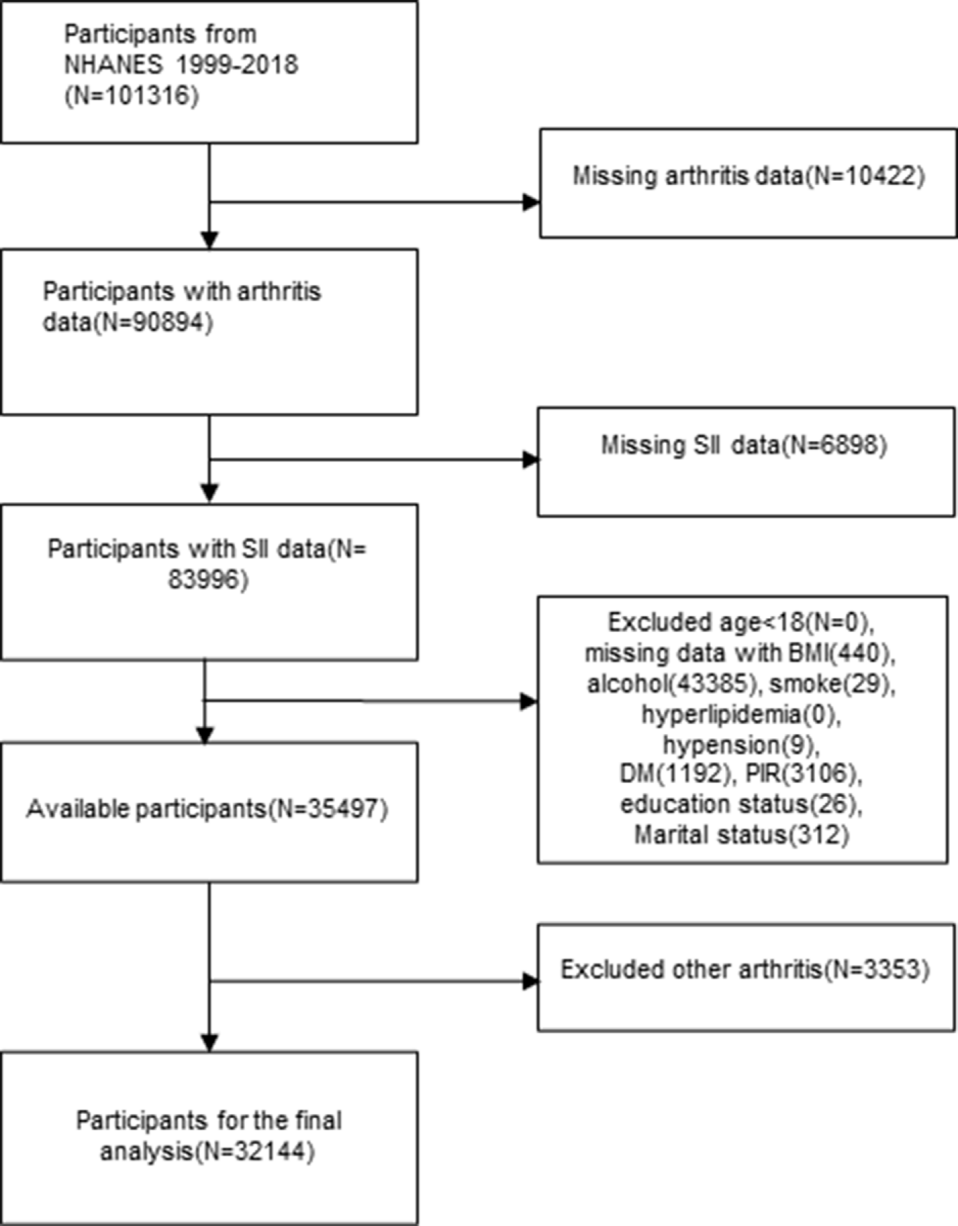
Flowchart of the screening of eligible population from the NHANES database 1999–2018. NHANES, National Health and Nutrition Examination Survey; SII, systemic immune-inflammation index; BMI, Body mass index; DM, diabetes mellitus.

In this particular study, we collected demographic data from 1999 to 2018, with a sample size of 101,316 people. After excluding those with incomplete data on arthritis (n ​= ​10,422), SII and SIRI (n ​= ​6898), those with incomplete variable survey data (n ​= ​48,499), and participants with other types of arthritis other than OA (n ​= ​3353), a total of 32,144 eligible participants were included in the final analysis. The flowchart illustrating this process is presented in Fig. 1. It is important to note that due to the availability of special weights for the period of 1999–2002, a weighting factor of 2/10 ​× ​wtmec4yr was applied to this 2-period, while all 8 cycles from 2003 to 2018 were weighted at 1/10 ​× ​wtmec2yr.

### 2.2 Assessment of SII, SIRI and OA

The necessary information for calculating SII and SIRI can be obtained from the complete blood samples collected during the participants' mobile Examination Center tests, provides blood cell, such as lymphocyte, neutrophil, and platelet counts for all participants and is expressed as ​× ​10^3^ ​cells/ml was expressed. The formula for calculating SII and SIRI is are as follows: SII = (P ​× ​N)/L [20]. SIRI = (N ​× ​M)/L [15]. The SII distribution exhibited a skewed pattern, prompting us to apply a logarithmic transformation to the SII values.

OA was diagnosed based on self-administered questionnaires completed by the participants during the clinical visit. The questionnaires comprised two questions: “Has a doctor or other health professional ever told you that you have had arthritis?”, “Which type of arthritis was it?”. Individuals with confirmed non-OA cases, including other types of arthritis, were excluded from the analysis, and participants who reported OA as their type of arthritis were selected for further investigation.

### 2.3 Covariate assessment

Covariates mainly included age, sex, race, Body Mass Index (BMI), marital status, Poverty income ratio (PIR), education status, alcohol consumption, smoking status, and the presence of hyperlipidemia and diabetes mellitus (DM). We divided participants aged 18 and above into two groups, using 50 years of age as the dividing point. This choice is based on the fact that individuals in this age range are in the later stages of their working lives and are more susceptible to developing OA, which can have a significant impact on long-term health and quality of life [22]. Race was categorized into four groups: non-Hispanic black, non-Hispanic white, Hispanic, and other race. Body Mass Index was given as < 25 ​kg/m^2^, 25–30 ​kg/m^2^ (overweight), >30 ​kg/m^2^ (obesity) as classification nodes. Marital status was classified into three categories: never married, married/living with partner, widowed/divorced/separated. The Poverty income ratio (PIR) was assigned at 130% of the federal poverty level to better assess participants' socioeconomic status. The PIR categories included PIR <1.3, 1.3–3.5, and >3.5. The education status was defined based on the completion of high school. The categories included high school, less than high school level and more than high school level. Alcohol consumption was classified as never, former, current (light, moderate, or heavy consumption**)** [23]. Smoking status was similar to the former, again with never, former, and now classification as classification criteria [24]. The presence of hyperlipidemia and diabetes was determined based on both self-reported questionnaire responses and examination data. Hyperlipidemia was assessed by participants' answers during the interview, indicating whether they had been diagnosed with hyperlipidemia (yes or no). According to the criteria of DM (Table S1), DM is divided into no, pre-diabetes and diabetes Hypertension was evaluated by considering whether participants had received a diagnosis of high blood pressure from a healthcare provider, were taking medication for hypertension, or had a blood pressure measurement exceeding 140/90 ​mmHg.

### 2.4 Statistical analysis

The NHANES database is investigated using complex multistage sampling. What's more, NHANES surveys are usually conducted every two years. Due to the impact of the novel coronavirus pandemic on survey visits at the end of 2019, the study population during the epidemic period was not included in the analysis. SII and SIRI values were categorized into quartiles (Q1, Q2, Q3, and Q4) based on ascending order, representing the lowest to highest values. T-tests, wilcoxon tests (continuous variables), and chi-square tests (categorical variables) were used to assess comparisons among different groups. Continuous variables are represented in either mean or quartile form according to the distribution characteristics. Categorical variables were presented as unweighted frequencies and weighted percentages.

**Fig. 2:**
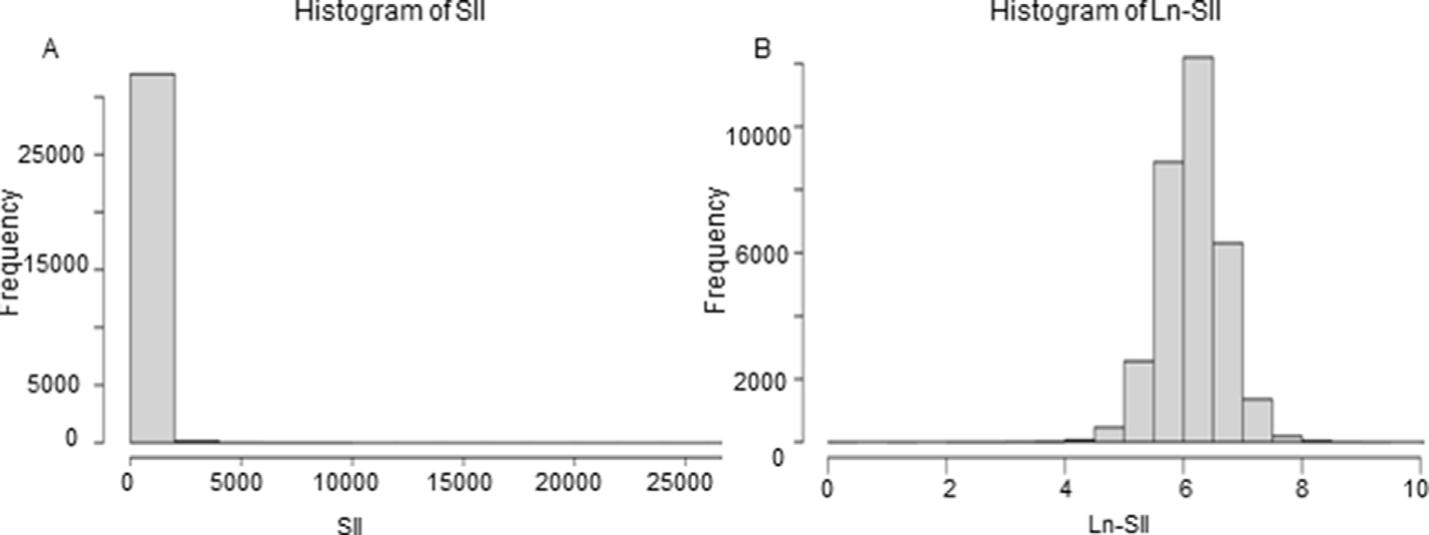
The map of untransformed SII distribution (A), and distribution of SII after ln-transformation (B). SII, systemic immune-inflammation index.

Furthermore, we constructed a regression model using multivariate logistic regression to examine the relationship between SII, SIRI and the occurrence of OA. In unadjusted Model, we used a crude model where no adjustments were made for other covariates. In Model 1, we adjusted for age and sex as covariables. In Model 2 we further adjusted for general situation and lifestyle habits, including BMI, PIR, education status, marital status, alcohol consumption, smoking status [25], hyperlipidemia, as well as DM. Collinearity diagnostics on SII, age, sex, race, BMI, PIR, education status, marital status, smoking status, alcohol consumption, hypertension, hyperlipidemia and DM were performed to prove no severe collinearity (both Variance Inflation Factor, VIF <2). When VIF >10, it is considered to indicate a severe collinearity. To further judge the nonlinear relationship between SII, SIRI and OA, we also employed a restricted cubic spline (RCS) fitting model. Due to the large span of SII values when exploring the nonlinear association between SII and periodontitis using RCS, SII was Ln-transformed in order to make it of the right order of magnitude to obtain reasonable logistic regression results, as detailed in Fig. 2.

In the final stage of our analysis, we perform stratification and interaction analysis. For the subgroup analyses, we adjusted for covariates including age, sex, BMI, PIR, alcohol consumption, and smoking status. All variables were adjusted, except the specific stratification factor used in each subgroup analysis. All statistical analyses were based on R (version 4.2.2) and NHANES R (version 0.9.4.3) packages. A significance threshold of 0.05 was used, and if a p-value was less than 0.05, it was considered statistically significant.

## 3 Results

### 3.1 Population characteristics

We included a total of 32,144 eligible participants from the NHANES database, covering the demographic data collected between 1999 and 2018. Among these participants, there were 16,515 males (51.38%) and 15,629 females (48.62%). The proportion of individuals diagnosed with OA in our study population was approximately 12.16% (Table S2). By utilizing hierarchical and cluster analysis methods, our inclusion population is estimated to represent approximately 145,667,006 individuals from the US non-institutionalized standard population during the corresponding time period.

As we can see, individuals with OA exhibited certain characteristics with higher proportions. These included a higher percentage of females (65.79%), age over 50 (80.82%), individuals of white race (84.67%), obesity (46.25%), individuals who were married/living with partner (64.67%), higher income levels (PIR >3.5; 46.86%),alcohol consumption (69.34%), hyperlipidemia (81.37%), hypertension (62.03%), and a higher proportion of SII and SIRI, please refer to Table 1. In the baseline table of population characteristics, it is observed that participants in the highest quartile of SII had a higher prevalence of certain characteristics. Specifically, they were more likely to have hyperlipidemia (71.03%), engage in alcohol consumption (76.40%), and have obesity (BMI >30 ​kg/m^2^ accounted for approximately 36.51%). Additionally, participants in this group tended to have relatively higher levels of education (61.38%). For more detailed information on these associations and other population characteristics, please refer to Table S2.

**Table 1:** Weighted characteristics of the study population based on OA.

Variable	Total	Non-OA	OA	*P*-value
Lymphocyte, 1000 ​cells/ul	2.00 (1.60,2.50)	2.00 (1.70,2.50)	1.90 (1.60,2.40)	<0.0001
Monocyte, 1000 ​cells/ul	0.50 (0.40,0.70)	0.50 (0.40,0.70)	0.60 (0.40,0.70)	<0.001
Neutrophils, 1000 ​cells/ul	4.00 (3.10,5.1)	4.00 (3.10,5.10)	4.10 (3.30,5.20)	0.011
Platelet, 1000 ​cells/ul	247.00 (210.00,290.00)	247.0 (211.00,291.00)	240.00 (202.00,287.00)	<0.0001
SII	484.77 (352.80,675.21)	482.17 (351.77,668.80)	503.25 (365.75,705.60)	<0.0001
SIRI	1.04 (0.73,1.50)	1.03 (0.72,1.48)	1.131 (0.81,1.65)	<0.0001
SIIQ				0.001
Q1	8036 (25.00)	7114 (22.63)	922 (21.20)	
Q2	8036 (25.00)	7152 (25.67)	884 (24.02)	
Q3	8036 (25.00)	7070 (26.05)	966 (25.18)	
Q4	8036 (25.00)	6901 (25.65)	1135 (29.60)	
SIRIQ				<0.0001
Q1	8041 (25.02)	7291 (22.80)	750 (17.16)	
Q2	8027 (24.97)	7141 (25.98)	886 (23.53)	
Q3	8056 (25.06)	7038 (26.03)	1018 (27.23)	
Q4	8020 (24.95)	6767 (25.20)	1253 (32.08)	
Sex				<0.0001
Female	15,629 (48.62)	13,130 (47.64)	2499 (65.79)	
Male	16,515 (51.38)	15,107 (52.36)	1408 (34.21)	
Age (years old)				<0.0001
>50	13,523 (42.07)	10,247 (30.33)	3276 (80.82)	
18-50	18,621 (57.93)	17,990 (69.67)	631 (19.19)	
Race				<0.0001
Black	6165 (19.18)	5612 (10.40)	553 (5.81)	
Mexican American and Other Hispanic	8189 (25.48)	7646 (14.67)	543 (4.84)	
other	2955 (9.19)	2733 (6.75)	222 (4.69)	
White	14,835 (46.15)	12,246 (68.18)	2589 (84.67)	
BMI (kg/m^2^)				<0.0001
<25	9980 (31.05)	9154 (33.92)	826 (21.52)	
>30	11,148 (34.68)	9335 (32.20)	1813 (46.25)	
25-30	11,016 (34.27)	9748 (33.88)	1268 (32.23)	
Marital status				<0.0001
Married/Living with Partner	19,737 (61.40)	17,431 (64.47)	2306 (64.67)	
Never married	6072 (18.89)	5808 (20.43)	264 (6.76)	
Widowed/Divorced/Separated	6335 (19.71)	4998 (15.10)	1337 (28.57)	
Poverty				0.002
1.3–3.5	12,204 (37.97)	10,661 (35.28)	1543 (36.34)	
<1.3	9240 (28.75)	8257 (20.12)	983 (16.80)	
>3.5	10,700 (33.29)	9319 (44.61)	1381 (46.86)	
Education status				0.373
high school	11,694 (36.38)	10,285 (33.50)	1409 (33.09)	
less than high school	3164 (9.84)	2845 (4.68)	319 (4.19)	
more than high school	17,286 (53.78)	15,107 (61.83)	2179 (62.72)	
Alcohol consumption				<0.0001
never	4360 (13.56)	3810 (10.68)	550 (11.50)	
former	5059 (15.74)	4158 (12.01)	901 (19.17)	
current	22,725 (70.70)	20,269 (77.30)	2456 (69.34)	
Smoking status				<0.0001
never	17,772 (55.29)	15,929 (56.17)	1843 (47.95)	
former	7606 (23.66)	6167 (22.00)	1439 (36.55)	
now	6766 (21.05)	6141 (21.83)	625 (15.50)	
Hyperlipidemia				<0.0001
no	9684 (30.13)	8973 (32.85)	711 (18.63)	
yes	22,460 (69.87)	19,264 (67.15)	3196 (81.37)	
Hypertension				<0.0001
no	19,816 (61.65)	18,531 (70.10)	1285 (37.97)	
yes	12,328 (38.35)	9706 (29.90)	2622 (62.03)	
DM				<0.0001
no	25,035 (77.88)	22,523 (84.26)	2512 (68.67)	
pre.DM	2279 (7.09)	1916 (6.19)	363 (9.36)	
DM	4830 (15.03)	3798 (9.55)	1032 (21.97)	

OA, osteoarthritis; SII, systemic immune-inflammation index; BMI, Body Mass Index; PIR, poverty-to-income ratio; DM, diabetes mellitus; pre.DM, which included Impaired fasting blood glucose and impaired glucose tolerance.

### 3.2 Association between SII, SIRI and OA

We proceeded to perform weighted multivariate logistic regression analysis (Table 2) and observed a potential association between SII and OA in unadjusted Model and Model 1. There was always a positive correlation between the highest quartile of SII and OA in the unadjusted model and adjusted model 1 (Unadjusted Model: OR ​= ​1.23, 95% CI ​= ​1.09–1.39, *P* ​< ​0.001; Model 1: OR ​= ​1.18, 95% CI ​= ​1.04–1.35, *P* ​= ​0.01). In Model 2, adjusting for all factors, a positive correlation between SII and OA risk was observed, although not statistically significant (Model 2: OR ​= ​1.13, 95% CI ​= ​0.99–1.29, *P* ​= ​0.07). Although the previous models demonstrated a consistent trend suggesting a positive association, the fully adjusted model revealed that the association between SII and OA prevalence did not reach statistical significance.

**Table 2:** Results of SII and OA multi-factor logistic regression.

SII	Unadjusted Model	*P*-value	Model 1	*P*-value	Model 2	*P*-value	SIRI	Unadjusted Model	*P*-value	Model 1	*P*-value	Model 2	*P*-value
	OR (95%CI)		OR (95%CI)		OR (95%CI)			OR (95%CI)		OR (95%CI)		OR (95%CI)	
Q1	ref		ref		ref		Q1	ref		ref		ref	
Q2	1.00 (0.88,1.13)	0.99	1.01 (0.89,1.16)	0.84	0.93 (0.81,1.06)	0.28	Q2	1.20 (1.05,1.37)	0.1	1.24 (1.08,1.43)	0.003	1.10 (0.95,1.27)	0.22
Q3	1.03 (0.91,1.17)	0.62	1.04 (0.92,1.18)	0.54	0.90 (0.78,1.03)	0.13	Q3	1.39 (1.23,1.57)	<0.0001	1.43 (1.25,1.63)	<0.0001	1.16 (1.01.1.38)	0.04
Q4	1.23 (1.11,1.37)	<0.001	1.22 (1.08,1.37)	0.001	1.01 (0.89,1.14)	0.91	Q4	1.69 (1.49,1.91)	<0.0001	1.70 (1.48,1.94)	<0.0001	1.28 (1.11,1.47)	0.001
P for trend		<0.0001		<0.001		0.580	P for trend		<0.0001		<0.0001		<0.001

OA, osteoarthritis; SII, systemic immune-inflammation index; SIRI, systemic Immune response Index.

Unadjusted Model, no covariates are adjusted.

Model 1, age and sex were adjusted.

Model 2, we adjusted for age, sex, BMI, PIR, eduation status, marital status (general condition), alcohol consumption, smoking status, hyperlipidemia, as well as diabetes mellitus (in terms of lifestyle habits).

The results demonstrated a dose-response relationship, where the morbidity of OA increased progressively with higher SII quartiles. Specifically, compared to the lowest quartile (SIIQ1), the odds of OA were elevated by 23% in the highest SII quartile (SIIQ4) of unadjusted Model, 22% in adjusted Model 1. However, the increase prevalence was minimal (1%) in the Model 2 (Table 2).

It is worth noting that SIRI and OA show a significant positive correlation. In the three models, the prevalence of OA gradually increased with the increase of SIRI, especially in Q3 and Q4. There was always a positive correlation between the highest quartile of SIRI and OA (Unadjusted Model: OR ​= ​1.69, 95% CI ​= ​1.49–1.91, *P* ​< ​0.0001; Model 1: OR ​= ​1.70, 95% CI ​= ​1.48–1.94, *P* ​< ​0.0001); Model 2: OR ​= ​1.28, 95% CI ​= ​1.11–1.47, *P* ​= ​0.001). In the logistic regression analysis model, we also included viral hepatitis (HBV, HCV) as covariates for analysis. The results indicated that even with the inclusion of viral hepatitis as covariates (Table S3), the relationship between SII and OA remained unchanged. Similarly, the relationship between SIRI and OA continued to show a positive correlation (P ​< ​0.05, OR>1).

### 3.3 RCS analysis

**Fig. 3:**
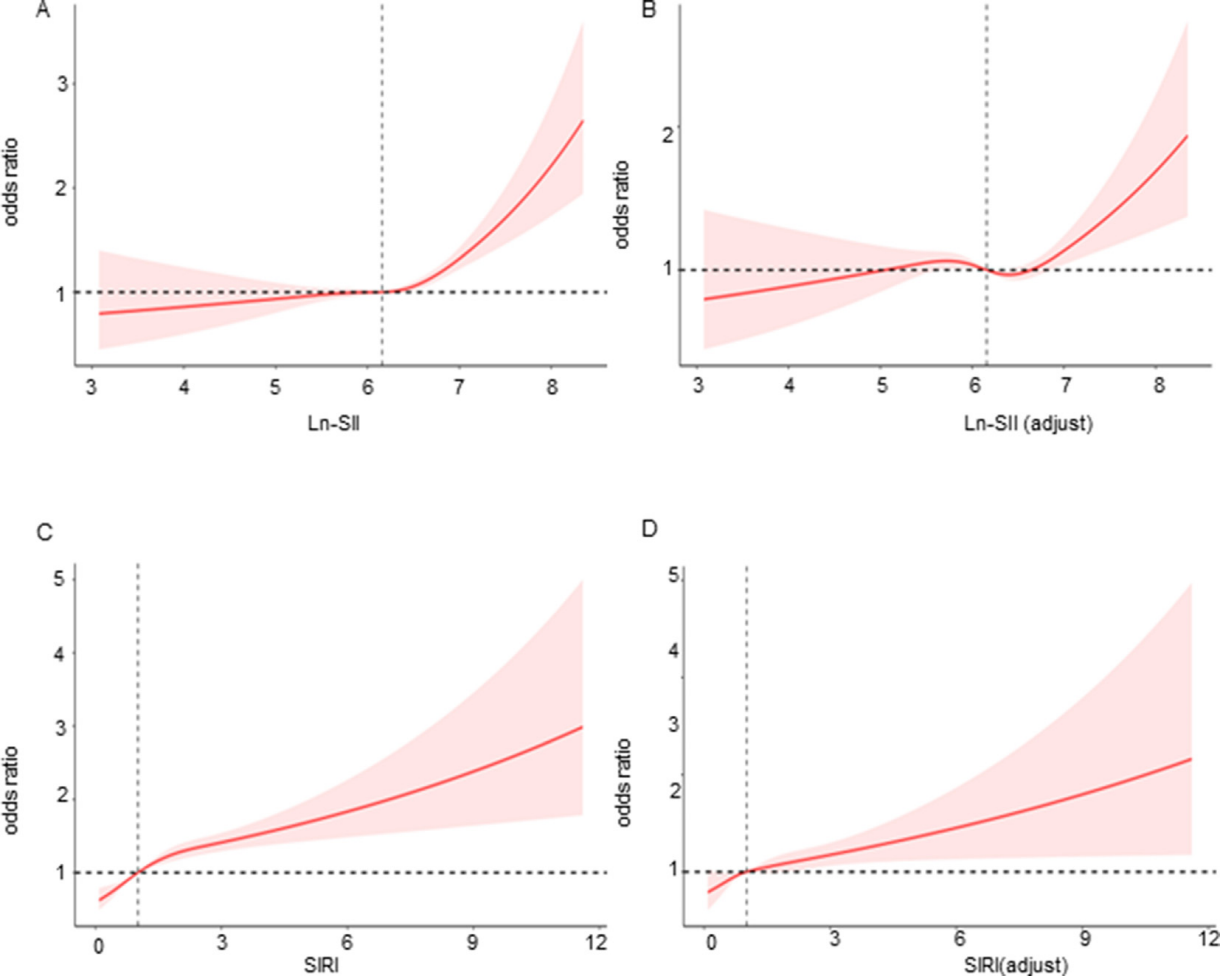
The non-adjusted nonlinearity relationship between ln-SII and OA (A). The full-adjusted nonlinearity relationship between ln-SII and OA (B). The non-adjusted nonlinearity relationship between SIRI and OA (C). The full-adjusted nonlinearity relationship between SIRI and OA (D). In the full-adjusted Model, age, sex, BMI, PIR, eduation status, marital status, alcohol consumption, smoking status, hyperlipidemia, as well as diabetes mellitus were adjusted. SII, systemic immune-inflammation index; OA, osteoarthritis; BMI, Body mass index; PIR, poverty to income ratio.

Surprisingly, we discovered a non-linear relationship between ln-SII and OA prevalence, both in the crude model and in Model 2, which was fully adjusted for the included confounders. The nonlinear analysis using RCSs revealed a statistically significant non-linear relationship (*p*-value: 4e-4 in the crude model; p-value: 0.0021 in Model 2). In contrast, there is no nonlinear correlation between SIRI and OA (Fig. 3). We identified a critical node at approximately ln-SII value of 6.39 (corresponding to SII ​= ​595.86). Before reaching this node, the relationship between ln-SII and OA appeared relatively smooth. However, once the ln-SII surpassed this critical node, the prevalence of OA exhibited a significant increase. This finding suggests that there may be a threshold effect where a certain level of ln-SII is associated with a notable elevation in OA.

### 3.4 Subgroup analysis

We further performed subgroup analyses to investigate whether the relationship between SII, SIRI and OA prevalence varied across different subgroups based on age, sex, ethnicity/Race, BMI, PIR, alcohol consumption, and smoking status. Table 3, Table 4 illustrates the results of these subgroup analyses. No interaction was found in the subgroup analysis.

**Table 3:** Association between SII and OA among subgroups.

Character	OR (95%CI)	*P*	*P* for interaction
Age			0.057
18–50	1.28 (1.05，1.58)	0.018	
>50	1.01 (0.91，1.12)	0.921	
**Sex**			0.276
Male	1.16 (1.01，1.33)	0.037	
Female	1.01 (0.90，1.12)	0.940	
**Race**			0.610
White	1.06 (0.97，1.17)	0.209	
Black	1.05 (0.85，1.30)	0.625	
Mexican American	1.17 (0.87，1.56)	0.293	
Other	0.77 (0.52，1.16)	0.215	
**PIR**			0.060
1.3–3.5	1.19 (1.03，1.37)	0.022	
<1.3	0.80 (0.73，1.00)	0.052	
>3.5	1.04 (0.89，1.21)	0.619	
**Alochol consumption**			0.457
never	0.91 (0.71，1.16)	0.419	
former	1.08 (0.90，1.29)	0.390	
current	1.07 (0.96，1.20)	0.207	
**Smoking status**			0.755
never	1.01 (0.89，1.15)	0.836	
former	1.10 (0.95，1.26)	0.201	
now	1.07 (0.87，1.32)	0.529	
**BMI**			0.183
25–30	0.99 (0.85，1.15)	0.863	
>30	1.02 (0.88，1.17)	0.822	
<25	1.19 (1.01，1.40)	0.042	

SII, systemic immune-inflammation index. OA, osteoarthritis. PIR, poverty-income ratio, BMI, Body Mass Index.

**Table 4:** Association between SIRI and OA among subgroups.

Character	OR (95%CI)	*P*	*P* for interaction
**Age**			0.400
18–50	1.10 (1.00,1.21)	0.048	
>50	1.10 (1.04,1.17)	<0.001	
**Sex**			0.693
Male	1.14 (1.07,1.22)	<0.001	
Female	1.07 (0.99,1.15)	0.078	
**Race**			0.243
White	1.09 (1.03,1.16)	0.002	
Black	1.07 (0.94，1.21)	0.296	
Mexican American	1.25 (1.10，1.43)	<0.001	
Other	0.96 (0.76，1.216)	0.739	
**PIR**			0.222
1.3–3.5	1.15 (1.06，1.23)	<0.001	
<1.3	1.01 (0.91，1.12)	0.914	
>3.5	1.09 (0.98，1.20)	0.099	
**Alochol consumption**			0.584
never	1.02 (0.88，1.18)	0.804	
former	1.13 (1.02，1.24)	0.016	
current	1.10 (1.04，1.18)	0.003	
**Smoking status**			0.628
never	1.10 (1.03，1.19)	0.008	
former	1.10 (1.02，1.19)	0.012	
now	1.08 (0.965，1.21)	0.175	
**BMI**			0.434
25–30	1.06 (0.98，1.17)	0.166	
>30	1.09 (0.99，1.20)	0.052	
<25	1.14 (1.05，1.24)	0.002	

SIRI, Systemic Immune Response Index. OA, osteoarthritis. PIR, poverty-income ratio, BMI, Body Mass Index.

## 4 Discussion

We included a total of 32,144 eligible participants from the NHANES database, covering the demographic data collected between 1999 and 2018. Among these participants, there were 16,515 males (51.38%) and 15,629 females (48.62%). The proportion of individuals diagnosed with OA in our study population was approximately 12.16% (Table S2). This Notably, the OA population exhibited relatively higher SII levels compared to the non-OA population.

The study revealed a significant association between SII and OA, indicating that the prevalence of OA increased as SII values increased in crude Model-Model 1. Furthermore, the RCS model, after adjusting for potential risk factors, demonstrated a significant nonlinear association between SII and OA prevalence. Specifically, when the SII value exceeded the threshold of 595.86, there was a notable increase in the prevalence rate of OA. In the three models, the prevalence of OA gradually increased with the increase of SIRI, especially in Q3 and Q4.

This study is the first to investigate the relationship between SII, SIRI and OA, shedding light on the potential connection between systemic inflammation and OA development. Previous studies have demonstrated the predictive power of SII in assessing disease activity and progression in rheumatoid arthritis, as well as its utility as an independent prognostic indicator for differentiating between psoriatic arthritis and rheumatoid arthritis [26,27].

The pathogenesis of OA is indeed multifactorial and involves various processes and mechanisms [28,29], including increased oxidative stress [30,31], AIM2 inflammasome activation [32], and extracellular matrix degradation [33,34]. This suggests a possible link between systemic inflammation and OA prevalence.

Indeed, evidence links psoriatic arthritis, rheumatoid arthritis, and inflammatory processes involving interleukin-1β-regulatory protein complexes and genes associated with inflammation. For instance, the gene CARD8-C10X (RS2043211) is implicated in psoriatic arthritis [32]. Inflammatory markers, including interleukins, may also contribute to OA's interstitial damage. Studies have associated elevated levels of specific markers like IL-2 and IL-1β with bone marrow lesions in knee OA [33]. These findings underscore the role of inflammation and metabolic factors in OA onset and progression [35]. Similarly, inflammatory markers, including interleukins, may also play a role in the interstitial injury associated with OA. Previous studies have linked serum inflammatory markers to the symptoms and structural changes observed in knee OA, with increased levels of certain inflammatory markers, such as IL-2 and IL-1β, being associated with the presence of bone marrow lesions [33]. These findings support the idea that changes in inflammatory and metabolic factors are related to OA development and progression.

OA involves subarticular cartilage damage, synovitis, and extracellular matrix damage, with significant leukocyte infiltration in the synovium, mainly macrophages and T lymphocytes [34]. Inflammatory chemokines drive leukocyte recruitment, trafficking, and activation, leading to the release of proinflammatory factors [35]. This likely plays a persistent role in OA-related synovitis [36].

These findings reinforce the idea that changes in inflammatory and metabolic factors are connected to OA development and progression. OA includes lesions like subarticular cartilage damage, synovitis, and extracellular matrix damage, along with significant leukocyte infiltration in the synovium, primarily macrophages and T lymphocytes [37]. Inflammatory chemokines promote leukocyte recruitment, trafficking, and activation, leading to the release of proinflammatory factors [38]. This likely contributes to sustained synovitis in OA.

OA is closely associated with adipose tissue, especially body fat, which, in conjunction with inflammation and metabolism, is thought to contribute to OA's development [39]. Inflammatory cells within adipose tissue can release proinflammatory factors, leading to systemic inflammation [40]. Importantly, individuals with obesity have distinct immune cell profiles compared to those with normal weight. They have increased numbers of lymphoblastoid cells like Th1 and B cells, elevated neutrophils [41], and reduced natural killer cells [42]. These findings underscore significant differences in immune cell composition between obese individuals and the general population.

Platelet volume, specifically mean platelet volume (MPV), is influenced by obesity and can indicate platelet function. MPV has been proposed as an inflammation marker, as suggested by scholar Balta [43,44]. Elevated MPV is linked to increased platelet activation and inflammation. Investigating the MPV-obesity relationship can offer more insights into the inflammatory processes in conditions like OA.

Kwon and colleagues discovered a positive association between platelet counts and OA prevalence in women over 50. Higher platelet counts (≥288 ​× ​10^3^/μl) correlated with an increased OA incidence [45]. This suggests that, like lymphocytes and neutrophils, platelets may contribute to OA development. Considering the possible link between platelet volume and OA, we examined the SII, which includes lymphocytes, neutrophils, and platelets, but found no significant association with OA in our study.

The study also investigated the role of race in the association between SII and OA risk. In multivariate logistic regression analysis, race was found to be a significant factor. Subgroup analysis revealed a positive link between white race and OA risk. However, there was no significant interaction effect between race and SII, indicating that race does not modify the relationship between SII and OA. Previous studies have analyzed the impact of race and gender on OA risk. Using logistic regression models, researchers have examined the relationship between race and gender and various OA-related parameters, such as Kellgren Lawrence grades and tibiofemoral joint space narrowing scores on knee radiographs. The results showed that among white Americans, males had significantly higher odds of radiographic Kellgren Lawrence grade progression and a greater likelihood of lateral JSN progression [46]. These findings highlight the potential influence of ethnicity and gender on OA and emphasize the need for further research to better understand these connections.

While some scholars like Liu initially regarded OA as solely a cartilage degenerative disease distinct from systemic immune conditions like rheumatoid arthritis [20], ongoing research has increasingly acknowledged the pivotal role of low-grade inflammation in OA development. The concept of the immune system's interaction with metabolic disorders in OA pathogenesis is gaining acceptance [47]. This evolving understanding has generated interest in anti-inflammatory therapies and sparked optimism for innovative OA treatments. Prior studies examining the association between the neutrophil-to-lymphocyte ratio (NLR) and early knee arthritis did not find a link between NLR and OA's clinical severity [48,49]. Inflammation indeed plays a crucial role in the development of cartilage degradation and OA. The release of inflammatory mediators accelerates the process of cartilage degradation, leading to vasodilation in the tissues surrounding the joints, resulting in swelling and pain. Cartilage degradation further increases friction between the joint bones, triggering arthritis. Similarly, osteoarthritis also accelerates cartilage degradation. Once osteoarthritis occurs, the inflammatory response and physiological changes around the joints expedite the process of cartilage degradation. This cyclical relationship between inflammation and cartilage degradation mutually exacerbates the development of OA. However, it's worth noting that these conclusions might have been influenced by limited sample sizes. Consequently, further investigation into the relationship between lymphocyte, neutrophil, and platelet counts and OA is warranted and merits attention.

This study has strengths that enhance our understanding of systemic inflammatory markers and OA. It challenges the misconception that SII is solely linked to autoimmune diseases and shows it may not be connected to other inflammatory conditions. This broadens our understanding of markers (SII and SIRI) potential applications and explores new avenues for anti-inflammatory approaches in OA prevention.

Our study has limitations. Firstly, our outcome variables relied on extensive questionnaires, potentially introducing recall bias and subjective errors. However, some studies have shown that self-reported data can be relatively reliable [50], and our study benefits from a large sample size, enhancing its credibility. Secondly, the generalizability of our results beyond the United States NHANES database should be considered. NHANES primarily comprises individuals of American ethnicity, and the relationship between SII and OA might differ in other ethnic groups. Further research in diverse populations is required to validate our findings externally. Additionally, our study was cross-sectional, limiting our ability to explore potential causal relationships between exposure and outcome. We also only adjusted for known confounders to a certain extent, and it's possible that other minor confounding factors influenced the outcomes.

## 5 Conclusion

Our study suggests a possible link among SII and SIRI levels with OA prevalence. Specifically, we observed that when SII exceeds 595.86, there may be a further increase in OA prevalence. This underscores SII and SIRI's potential as a valuable inflammatory marker for gaining insights into OA's pathogenesis and prognosis.

## Funding

This work was funded by Chengdu Science and Technology Bureau Project (2019-YF05-00498-SN) and Science and Technology Department of Sichuan Province Project (2021YJ0170).

## Author contributions

Study design: Yan Xue, Cheng Chang, Yajun Chen, Lang Jia, Han Wang, Zaoyang Liu, Jiang Xie

Data collection and data analysis: Yan Xue, Cheng Chang, Yajun Chen, Lang Jia

Drafting manuscript: Yan Xue, Cheng Chang, Yajun Chen

All authors take responsibility for the integrity of the data analysis. All authors have read and approved the article.

## Data availability statement

The NHANES dataset is publicly available online, accessible at cdc. gov/nchs/nhanes/index.htm.

## Ethics approval

All the data used in our study were obtained from the National Health and Nutrition Examination Survey (NHANES). NHANES is a nationally representative cross-sectional study conducted under the direction of the National Center for Health Statistics (NCHS) to assess the health and nutrition status of the non-institutionalized population of the United States using a complex, multistage, and probabilistic sampling design. All of the surveys were authorized by the NCHS Ethics Review Board before being conducted, and all participants signed informed consent forms. More information is available at http://www.cdc.gov/nchs/nhanes/.

## Conflicts of interest

None of the authors have any potential conflicts of interest.
