# Supplementary material for: Association between the immune-inflammation indicators and osteoarthritis - NHANES 1999–2018
Source: Osteoarthr Cartil Open. 2024 Feb 29;7(1):100453. doi: 10.1016/j.ocarto.2024.100453 (PMC11720436; doi:10.1016/j.ocarto.2024.100453)
Supplement: Multimedia component 2 [file mmc2.docx]

Table S1. Weighted demographic characteristics of all participants.

| Variable | Total | Q1 | Q2 | Q3 | Q4 | *P*-value |
| --- | --- | --- | --- | --- | --- | --- |
| Lymphocyte, 1000cells/ul | 2.00 (1.6,2.5) | 2.30(1.90,2.80) | 2.10(1.70,2.60) | 2.00(1.60,2.40) | 1.80(1.40,2.20) | < 0.0001 |
| Monocyte, 1000cells/ul | 0.50 (0.40,0.70) | 0.50(0.40,0.60) | 0.50(0.40,0.60) | 0.50(0.40,0.70) | 0.60(0.500,0.70) | < 0.0001 |
| Neutrophils, 1000cells/ul | 4.00 (3.10,5.10) | 2.80(2.30,3.50) | 3.60(3.10,4.30) | 4.30(3.60,5.10) | 5.40(4.50,6.70) | < 0.0001 |
| Platelet, 1000cells/ul | 247.00 (210.00,290.00) | 208.00 (179.00,239.00) | 235.00(207.00,269.00) | 258.00(226.00,296.00) | 289.00(248.00,338.00) | < 0.0001 |
| Sex |  |  |  |  |  | < 0.0001 |
| Female | 15629(48.62) | 3454(43.42) | 3784(47.55) | 4057(50.76) | 4334(56.91) |  |
| Male | 16515(51.38) | 4586(56.58) | 4253(52.45) | 3973(49.24) | 3703(43.09) |  |
| Age, years old |  |  |  |  |  | 0.29 |
| >50 | 13523(42.07) | 3478(37.35) | 3332(35.80) | 3268(36.16) | 3445(37.26) |  |
| 18-50 | 18621(57.93) | 4562(62.65) | 4705(64.20) | 4762(63.84) | 4592(62.74) |  |
| Race |  |  |  |  |  | < 0.0001 |
| Black | 6165(19.18) | 2546(17.76) | 1444( 9.05) | 1151( 7.25) | 1024( 6.34) |  |
| Other Hispanic | 8189(25.48) | 1827(13.10) | 2135(13.77) | 2195(13.86) | 2032(13.02) |  |
| other | 2955(9.19) | 863(7.50) | 800(6.73) | 694(6.43) | 598(5.47) |  |
| White | 14835(46.15) | 2804(61.65) | 3658(70.46) | 3990(72.47) | 4383(75.18) |  |
| BMI, kg/m^2^ |  |  |  |  |  | < 0.0001 |
| <25 | 9980(31.05) | 2705(35.72) | 2468(32.64) | 2362(30.19) | 2445(31.42) |  |
| >30 | 11148(34.68) | 2485(29.38) | 2697(31.85) | 2944(36.51) | 3022(37.38) |  |
| 25-30 | 11016(34.27) | 2850(34.90) | 2872(35.51) | 2724(33.30) | 2570(31.20) |  |
| Marital status |  |  |  |  |  | < 0.0001 |
| Married/Living with Partner | 19737(61.40) | 4960(65.65) | 5107(66.86) | 4982(64.97) | 4688(60.73) |  |
| Never married | 6072(18.89) | 1605(19.80) | 1443(18.09) | 1493(18.19) | 1531(18.94) |  |
| Widowed/Divorced/Separated | 6335(19.71) | 1475(14.55) | 1487(15.05) | 1555(16.84) | 1818(20.33) |  |
| PIR |  |  |  |  |  | 0.088 |
| 1.3-3.5 | 12204(37.97) | 3050(35.52) | 3030(35.10) | 3012(34.85) | 3112(36.18) |  |
| <1.3 | 9240(28.75) | 2358(20.43) | 2268(19.16) | 2272(19.00) | 2342(20.30) |  |
| >3.5 | 10700(33.29) | 2632(44.05) | 2739(45.74) | 2746(46.15) | 2583(43.52) |  |
| Education level |  |  |  |  |  | < 0.001 |
| high school | 7285(22.66) | 1741(21.65) | 1753(22.29) | 1870(24.12) | 1921(24.87) |  |
| less than high school | 7573(23.56) | 1923(15.43) | 1872(14.26) | 1894(14.50) | 1884(14.96) |  |
| more than high school | 17286(53.78) | 4376(62.92) | 4412(63.45) | 4266(61.38) | 4232(60.17) |  |
| Alcohol consumption |  |  |  |  |  | < 0.0001 |
| never | 4360(13.56) | 1158(11.84) | 1116(10.96) | 1052(10.38) | 1034(10.11) |  |
| former | 5059(15.74) | 1237(11.67) | 1184(11.78) | 1231(13.22) | 1407(14.75) |  |
| current | 22725(70.70) | 5645(76.49) | 5737(77.26) | 5747(76.40) | 5596(75.14) |  |
| Smoking status |  |  |  |  |  | < 0.0001 |
| never | 17772(55.29) | 4652(57.56) | 4544(56.40) | 4465(55.94) | 4111(51.0) |  |
| former | 7606(23.66) | 1819(23.64) | 1895(23.87) | 1859(22.99) | 2033(24.73) |  |
| now | 6766(21.05) | 1569(18.81) | 1598(19.73) | 1706(21.07) | 1893(24.22) |  |
| Hyperlipidemia |  |  |  |  |  | < 0.0001 |
| no | 9684(30.13) | 2717(35.23) | 2439(31.71) | 2267(28.99) | 2261(28.97) |  |
| yes | 22460(69.87) | 5323(64.78) | 5598(68.29) | 5763(71.01) | 5776(71.03) |  |
| Hypertension |  |  |  |  |  | < 0.0001 |
| no | 19816(61.65) | 5056(68.10) | 5132(68.45) | 4958(65.58) | 4670(62.57) |  |
| yes | 12328(38.35) | 2984(31.90) | 2905(31.55) | 3072(34.42) | 3367(37.43) |  |
| DM |  |  |  |  |  | 0.004 |
| no | 25035(77.88) | 6320(83.20) | 6297(83.14) | 6299(82.50) | 6119(80.56) |  |
| pre.DM | 2279(7.09) | 555(6.60) | 563(6.31) | 564(6.41) | 597(7.01) |  |
| DM | 4830(15.03) | 1165(10.20) | 1177(10.55) | 1167(11.08) | 1321(12.43) |  |
| OA |  |  |  |  |  | 0.001 |
| no | 28237(87.85) | 7118(88.23) | 7153(88.24) | 7064(87.89) | 6902(85.88) |  |
| yes | 3907(12.16) | 922(11.77) | 884(11.76) | 966(12.11) | 1135(14.12) |  |

OA, osteoarthritis; SII, systemic immune-inflammation index; BMI, Body Mass Index; PIR, poverty-to-income ratio; DM, diabetes mellitus; pre.DM, which included Impaired fasting blood glucose and impaired glucose tolerance.
