# Supplementary material for: Association between the immune-inflammation indicators and osteoarthritis - NHANES 1999–2018
Source: Osteoarthr Cartil Open. 2024 Feb 29;7(1):100453. doi: 10.1016/j.ocarto.2024.100453 (PMC11720436; doi:10.1016/j.ocarto.2024.100453)
Supplement: Multimedia component 3 [file mmc3.docx]

Table S3. The table depicting the relationship between SII, SIRI, and OA after adjusting for hepatitis infection.

| **SII** | Model 3 |  | **SIRI** | Model 3 |  |
| --- | --- | --- | --- | --- | --- |
|  | OR (95%CI) | *P*-value |  | OR (95%CI) | *P*-value |
| Q1 | ref |  | Q1 | ref |  |
| Q2 | 0.94(0.82,1.07) | 0.34 | Q2 | 1.10(0.94,1.28) | 0.22 |
| Q3 | 0.92(0.80,1.06) | 0.92 | Q3 | 1.17(1.02,1.35) | 0.03 |
| Q4 | 1.02(0.90,1.16) | 0.70 | Q4 | 1.30(1.12,1.50) | <0.001 |
| P for trend |  | *0.419* | P for trend |  | ***<0.001*** |

Model 3, we adjusted for age, sex, BMI, PIR, eduation status, marital status (general condition), alcohol consumption, smoking status, hyperlipidemia, diabetes mellitus as well as **viral hepatitis.**
